# Pharmacological Inhibition of NLRP3 Inflammasome Attenuates Myocardial Ischemia/Reperfusion Injury by Activation of RISK and Mitochondrial Pathways

**DOI:** 10.1155/2016/5271251

**Published:** 2016-12-08

**Authors:** Raffaella Mastrocola, Claudia Penna, Francesca Tullio, Saveria Femminò, Debora Nigro, Fausto Chiazza, Loredana Serpe, Debora Collotta, Giuseppe Alloatti, Mattia Cocco, Massimo Bertinaria, Pasquale Pagliaro, Manuela Aragno, Massimo Collino

**Affiliations:** ^1^Department of Clinical and Biological Sciences, University of Turin, Torino, Italy; ^2^Department of Drug Science and Technology, University of Turin, Torino, Italy; ^3^Department of Life Sciences and Systems Biology, University of Turin, Torino, Italy

## Abstract

Although the nucleotide-binding oligomerization domain- (NOD-) like receptor pyrin domain containing 3 (NLRP3) inflammasome has been recently detected in the heart, its role in cardiac ischemia/reperfusion (IR) is still controversial. Here, we investigate whether a pharmacological modulation of NLRP3 inflammasome exerted protective effects in an* ex vivo* model of IR injury. Isolated hearts from male Wistar rats (5-6 months old) underwent ischemia (30 min) followed by reperfusion (20 or 60 min) with and without pretreatment with the recently synthetized NLRP3 inflammasome inhibitor INF4E (50 *μ*M, 20 min before ischemia). INF4E exerted protection against myocardial IR, shown by a significant reduction in infarct size and lactate dehydrogenase release and improvement in postischemic left ventricular pressure. The formation of the NLRP3 inflammasome complex was induced by myocardial IR and attenuated by INF4E in a time-dependent way. Interestingly, the hearts of the INF4E-pretreated animals displayed a marked improvement of the protective RISK pathway and this effect was associated increase in expression of markers of mitochondrial oxidative phosphorylation. Our results demonstrate for the first time that INF4E protected against the IR-induced myocardial injury and dysfunction, by a mechanism that involves inhibition of the NLRP3 inflammasome, resulting in the activation of the prosurvival RISK pathway and improvement in mitochondrial function.

## 1. Introduction

Ischemic heart disease is one of the main culprits of illness and death [1, 2]. The main outcome of a transient cardiac ischemia is the progressive decline of the left ventricle contractile function, frequently paralleled by impairment of the mitochondrial energy metabolism [3, 4]. During the reperfusion phase the sudden mitochondrial oxygen overload induces oxidative stress and further worsens the metabolic derangement [4], thus paradoxically exacerbating myocardial injury and inducing pyroptosis [2, 5]. Pyroptosis is a caspase-1-dependent process leading to cell lysis, which has been demonstrated to be strongly regulated by the multiprotein platform complex nucleotide-binding oligomerization domain- (NOD-) like receptor pyrin domain containing 3 (NLRP3) inflammasome. The NLRP3 inflammasome comprises (a) NLRP3, (b) an apoptosis-associated speck-like protein containing a caspase activation recruitment domain (ASC), and (c) procaspase-1. In response to a wide range of danger signals, including oxygen-free radicals, K^+^ efflux, or mitochondrial stress [6–8], NLRP3 recruits the adaptor protein ASC which in turn interacts with procaspase-1. Inflammasome oligomerization promotes the autocatalytic activation of procaspase-1 and the processing of prointerleukin- (IL-) 1*β* [2]. More recently, a new protein has been identified as member of the NLRP3 inflammasome complex, the Gasdermin D (GSDMD), which is recruited with kinetics similar to those required for caspase-1 activation. The proteolytic cleavage of GSDMD by caspase-1 detaches its N-terminal fragment, which contributes to mediate IL-1*β* secretion and pyroptosis [9]. Since NLRP3 is detectable in many cardiac cell types, including cardiofibroblasts (the most important cell type in the heart in terms of number of cells) and cardiomyocytes (the most important cell type in terms of cell volumes), it is likely that it may play a pivotal role in acute myocardial infarction [10, 11]. Indeed, we and others have shown that NLRP3 is upregulated by ischemia/reperfusion (IR) injury and its myocardial activation is exacerbated by metabolic derangements [12, 13]. Interestingly, genetic modulation of NLRP3 has been reported to reduce myocardial infarct sizes upon IR [13]. However, a very recent study failed to find any role of NLRP3 in determining myocardial IR injury [14] and another investigation supported cardioprotective effects due to NLRP3 inflammasome activation, thus highlighting that the interpretation of NLRP3 inflammasome role in myocardial IR injury is far from clear. Nevertheless, a cross-talk between NLRP3 and mitochondria, the main player of IR injury, has been described, with NLRP3 being able to sense the presence of reactive oxygen species (ROS) produced by normal or dysfunctional mitochondria [15]. Thus, the present study aimed to investigate the effects of a newly synthesized NLRP3 inflammasome inhibitor, named INF4E [16], in an* ex vivo* model of myocardial IR injury. We deepened our investigation evaluating its ability, in the rat heart, (i) to interfere with the IR-induced NLRP3 inflammasome activation and pyroptotic cascade and (ii) to improve the mitochondrial metabolic response to IR insult.

## 2. Materials and Methods

### 2.1. INF4E Preparation

INF4E was dissolved at 200 mM concentration in DMSO. Stock solution was then diluted at a final concentration of 50 *µ*M in the perfusion buffer (see below). The description of the synthesis and the specificity of the inhibitor is included in the Supplemental Material (available online at http://dx.doi.org/10.1155/2016/5271251), according to previous publications [16, 17].

### 2.2. Animals Protocol and* Ex Vivo* Ischemia/Reperfusion (IR) Injury

Male Wistar rats (Harlan Laboratories, Udine, Italy) 5-6 months old, reaching a body weight of 450–550 g, were cared in compliance with the European Directive 2010/63/EU on the protection of animals used for scientific purposes. The animal protocols followed in this study were approved by the local “Animal Use and Care Committee.” After one week of quarantine, with drink and food* ad libitum*, rats were anesthetized and killed. The hearts were rapidly perfused. A constant flow was maintained to obtain a typical coronary perfusion pressure of about 80 mm Hg by the Langendorff technique with Krebs-Henseleit bicarbonate buffer containing (mM) NaCl 118, NaHCO_3_ 25, KCl 4.7, KH_2_PO_4_ 1.2, MgSO_4_ 1.2, CaCl_2_ 1.25, and Glucose 11. The buffer was gassed with 95% O_2_ : 5% CO_2_. The temperature of the perfusion system was maintained at 37°C. The hearts underwent 30 min stabilization and then were exposed to 30 min of global no-flow, normothermic ischemia followed by a period of 20 or 60 min of reperfusion. Hearts from a subgroup of rats (IR+INF4E) were pretreated with 50 *μ*M INF4E in the perfusate for 20 min before ischemia (after the first 10 min of stabilization). Hearts from sham animals were exposed to 60 min perfusion only and served as reference group in Western blot analysis.

The hearts were electrically paced at 280–300 bpm and kept in a temperature-controlled chamber (37°C). Pacing was stopped at the beginning of the ischemia and restarted after the third min of reperfusion. Left ventricular pressure (LVP) and coronary perfusion pressure (CPP) were recorded and monitored with two electromanometers placed within the left ventricle and along the perfusion line, respectively. Coronary flow, CPP, and LVP were used as indices of preparation conditions. Moreover, end diastolic LVP was recorded as index of contracture development during I/R and developed ventricular pressure as index of contractile activity throughout the experiment using PowerLab data acquisition system and analyzed using Chart software (ADInstruments, Oxford, UK).

The perfusate flowing out of the heart was collected for measurement of lactate dehydrogenase (LDH) release 5 min immediately before ischemia and for the entire reperfusion period. To assess the conditions of experimental preparation the coronary flow rate was determined by the amount of perfusate measured in a specific time period. The heart was then cut in two parts by a coronal section (perpendicular to the long axis). The apical part (less than 1/3 of ventricular mass) was used for molecular analysis and, thus, frozen rapidly in liquid nitrogen and stored at −80°C. Infarct size assessment was performed by using the basal part of ventricle.

### 2.3. Measurement of the Infarct Size

Infarct mass was evaluated at the end of the reperfusion with the nitro-blue-tetrazolium (NBT) technique by using a gravimetric method [18]. The basal part of the ventricles was dissected by transverse sections into two/ three slices, which were incubated for 20 min with a solution of NBT (0.1%) in phosphate buffer. Two independent and blind observers carefully separated and then weighted both stained and unstained tissues. Total mass of necrosis was then calculated and expressed as percentage of ventricular mass. Since the ischemia was global and since we analyzed only the basal part of the ventricles the necrotic mass was expressed as a percentage of the analyzed ischemic tissue.

### 2.4. LDH Assay

Spectrophotometric analysis at 340 nm was performed on the collected perfusion effluent to measure LDH released from the heart.

### 2.5. Preparation of Tissue Extracts

Total proteins extracts were obtained from 10% (w/v) apex homogenates in RIPA buffer (0.5% Nonidet P-40, 0.5% sodium deoxycholate, 0.1% SDS, 10 mmol/l EDTA, and protease inhibitors) as previously described [19]. Protein concentrations were measured by Bradford assay (BioRad, Hercules, CA, USA) and samples were then stored at −80°C for subsequent analysis.

### 2.6. Determination of IL-1*β* in Hearts Homogenates

Commercially available ELISA kit (R&D Systems, Abingdon, UK) was used to measure concentrations of IL-1*β* in tissue homogenates, according to the manufacturer's instructions.

### 2.7. Western Blot Analysis

Total proteins extracts were separated by SDS-PAGE and blotted to nitrocellulose membrane (GE-Healthcare Europe, Milano, Italy). Membranes were incubated with rabbit anti-NLRP3 (Abcam, Cambridge, UK), rabbit anti-caspase-1 (Santa Cruz Biotechnology, Dallas, TX, USA), mouse anti-GSDMDC1 (Santa Cruz Biotechnology), rabbit anti-IL-1*β* (Santa Cruz Biotechnology), rabbit anti-caspase-1 (Santa Cruz Biotechnology, Dallas, TX, USA), mouse anti-Tyr^204^ ERK1/2 (Cell Signaling Technology), rabbit anti-total ERK1/2 (Cell Signaling Technology), mouse anti-Ser^473^ Akt (Cell Signaling Technology), rabbit anti-total Akt (Cell Signaling Technology), rabbit anti-Ser^9^ GSK-3*β* (Abcam, Cambridge, UK), anti-total GSK-3*β* (Cell Signaling Technology), rabbit anti-mitochondrial transcription factor A (mtTFA) (Novus Biologicals, Cambridge, UK), mouse anti-nuclear respiratory factor-1 (NRF-1) (Santa Cruz Biotechnology), and mouse anti-sarcomeric mitochondrial creatine kinase (sMtCK) (Santa Cruz Biotechnology) and then probed with proper HRP-conjugated secondary antibodies (BioRad). Clarity Western ECL substrate (BioRad) was used for protein detection and quantification was performed by densitometric analysis (Quantity-One, Bio-Rad software). Data were normalized according to the related antitubulin densitometric values.

### 2.8. Real-Time PCR

Total RNA was extracted from heart samples using the AllPrep® DNA/RNA/protein kit (Qiagen, Hilden, Germany), according to the manufacture instructions. The total RNA concentration (*μ*g/mL) was determined by the fluorometer Qubit and the Quant-iT™ RNA Assay Kit (Invitrogen, Milano, Italy). A total of 500 ng of RNA was reverse-transcribed using QuantiTect Reverse Transcription Kit (Qiagen). The synthesized cDNA was used for real-time polymerase chain reaction (RT-PCR). The cDNA was amplified by real-time PCR using SsoFast™ EvaGreen (Bio-Rad,) and primers specific for cytokine IL-1*β* (Mm_Il1b_2_SG, cat. number QT01048355, Qiagen). The PCR reaction was performed at 95°C for 30 s followed by 40 cycles of 95°C for 5 s, 55°C for 10 s. All samples were run in duplicate. At least two nontemplate controls were included in all PCR. The transcript of the reference gene ribosomal RNA 18S (Mm_Rn18s_3_SG, cat. number QT02448075, Qiagen) was used to normalize mRNA data, and the quantification data analyses were performed by using the Bio-Rad CFX Manager Software, version 1.6 (Bio-Rad) according to the manufacturer's instructions.

### 2.9. Materials

Compounds here used were obtained from the Sigma-Aldrich Company Ltd., unless otherwise stated.

### 2.10. Statistical Analysis

Data described in the text and figures are presented as means ± standard error of the mean (s.e.m.) of *n* observations, where *n* represents the number of animals studied. Statistical analysis was performed using ANOVA test followed by Bonferroni's posttest. A *P* value of less than 0.05 was considered to be statistically significant.

## 3. Results

### 3.1. INF4E Pretreatment Limits Infarct Size and Improves Contractility Recovery

**Figure 1:**
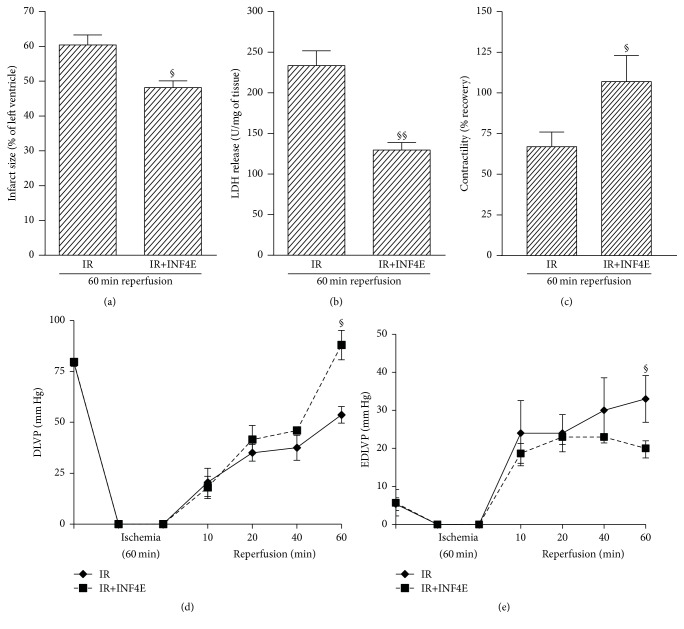
Infarct area, LDH release, and contractility recovery in hearts from rats exposed to 30 min of ischemia plus 60 min of reperfusion, pretreated or not with 50 *µ*M INF4E in the perfusate 20 min before ischemia. (a): infarct size after IR exposition is expressed as a percentage of ischemic tissue (% IS/IT). (b): LDH release in the perfusion effluent during the IR was expressed as units per mg of wet tissue weight. (c)–(e): monitoring of left ventricular pressure (LVP) was used to assess the contractility response to ischemia reperfusion injury. (c) shows the percentage of contractility recovered at the end of 60 min reperfusion. (d) and (e) show the entire time-course of developed LVP (DLVP) and end diastolic LVP (EDLVP), respectively. Data are means of 6 rats ± SEM. ^§^ *P* < 0.05 versus IR. ^§§^ *P* < 0.01 versus IR.

Rat hearts exposed to a 30 min global ischemia and 60 min reperfusion developed a 60 ± 3% infarct size in the basal portion of ventricle evaluated by NBT staining. When hearts were pretreated (20 min prior to ischemia) with INF4E in the perfusate, the infarct size was significantly reduced compared to untreated hearts (IR) (Figure 1(a)). Importantly, LDH release in the perfusate was almost halved by drug treatment (Figure 1(b)). In addition, the INF4E pretreated hearts showed a twofold increase in contractile recovery after IR, as assessed by left ventricular pressure monitoring (Figure 1(c)).

Of note, IR caused an impairment of the mechanical performance, as evidenced by the dramatic reduction of developed LVP (DLVP) immediately after ischemia and the incomplete recovery during reperfusion. In fact, developed LVP fell from about 80 mmHg in the preischemic condition to about 25 mmHg after 10 min of reperfusion and then recovered to only 50 mmHg at 60 min reperfusion, thus reaching only less than 65% of the preischemic value (Figures 1(c) and 1(d)). The pretreatment with the NLRP3 inhibitor did not modify the recovery of DLVP at early reperfusion time (10 and 20 min), whereas an improvement starts to be detectable after 40 min, reaching values up to 85 mmHg at 60 min, corresponding to over 100% of the preischemic value at the end of reperfusion. The end diastolic LVP (EDLVP) was about 5 mmHg during stabilization; then, the 30 min global ischemia and the subsequent reperfusion caused a sustained increase of this parameter in both the IR and IR+INF4E groups (Figure 1(e)). Actually, an increase of EDLVP was appreciated immediately after the end of ischemia and continued during the first 20 min of reperfusion. Then EDLVP further increased in the IR group reaching values over 30 mm Hg, while in the group pretreated with the NLRP3 inhibitor it decreased progressively to about 20 mmHg after 1 hour of reperfusion. The increase in EDLVP was significantly (*P* < 0.05) attenuated by the NLRP3 inhibitor only at 60 min reperfusion.

### 3.2. INF4E Pretreatment Prevents NLRP3 Inflammasome Activation and Downstream Signaling

**Figure 2:**
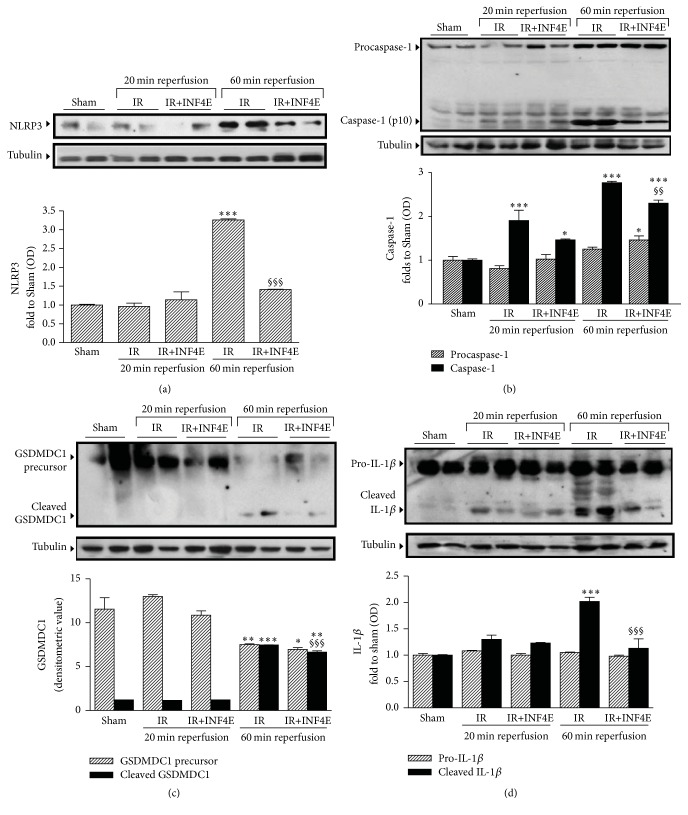
Inflammasome expression and activation in the rat heart exposed to 30 min ischemia plus 20 or 60 min reperfusion, pretreated or not with INF4E. Representative Western blotting showing cardiac levels of NLRP3 and of downstream activation of caspase-1, GSDMDC1, and IL-1*β* cleavage assessed on heart extracts. Histograms report densitometric analysis normalized for the corresponding tubulin content. Data are means of 6 rats ± SEM. ^*∗*^ *P* < 0.05, ^*∗∗*^ *P* < 0.01, and ^*∗∗∗*^ *P* < 0.001 versus Sham; ^§§^ *P* < 0.01, ^§§§^ *P* < 0.001 versus IR.

**Figure 3:**
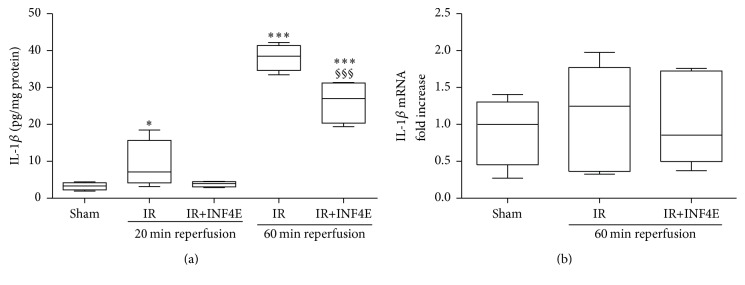
IL-1*β* concentrations evaluated by ELISA (a) and mRNA levels of IL-1*β* measured by RT-PCR (b) in extracts of rat hearts exposed to 30 min ischemia plus 20 or 60 min reperfusion, pretreated or not with INF4E. Data are means of 6 rats ± SEM. ^*∗*^ *P* < 0.05, ^*∗∗∗*^ *P* < 0.001 versus Sham; ^§§§^ *P* < 0.001 versus IR.

To confirm the ability of INF4E to interfere with NLRP3 inflammasome complex formation and activation in our experimental model, the expression level and the activation of the downstream signaling of NLRP3 inflammasome were assessed by Western blotting analysis in protein extracts obtained from the apical portion of hearts pretreated or not with INF4E and exposed to IR (Figure 2). Besides, rat hearts were exposed to two different periods of reperfusion, short and long (20 min and 60 min, resp.) to better elucidate the kinetics of the pharmacological modulation of NLRP3 inflammasome and related pathways. As shown in Figure 2, the protein level of NLRP3 was increased in a time-dependent manner, reaching statistical significance only after the 60 min reperfusion. Notably, the INF4E pretreatment effectively reduced its upregulation. Western blotting analysis with an antibody recognizing the C-terminal region of caspase-1 allowed the identification of two bands corresponding to the procaspase-1 and the cleaved active p10 subunit of caspase-1. After 20 min of reperfusion the active form of caspase-1 was markedly increased, without significant changes in the expression of procaspase-1, although the INF4E-pretreated hearts showed a slight nonsignificant trend towards reduced procaspase cleavage. After 60 min of reperfusion, both precursor and active forms of caspase-1 were dramatically increased in the untreated IR group, whereas the INF4E-pretreatment significantly prevented procaspase cleavage. As shown in Figure 2(c), caspase-1 activation is associated with cleavage of the GSDMDC1 component of the inflammasome platform, detectable only after the longest reperfusion time. Interestingly, INF4E pretreatment significantly reduced GSDMCD1 cleavage. Accordingly, the protein levels of the active form of IL-1*β* showed a robust increase after the 60 min reperfusion, as demonstrated by Western blot analysis (Figure 2) and confirmed by ELISA (Figure 3(a)). The INF4E pretreatment prevented the slight increase in IL-1*β* production due to 20 min of reperfusion and strongly reduced the massive IL-1*β* release recorded after 60 min of reperfusion (Figures 2 and 3(a)). Interestingly, neither the IR injury nor the drug treatment affected the mRNA levels of IL-1*β* (Figure 3(b)), thus confirming a selective effect of INF4E on NLRP3 inflammasome-dependent IL-1*β* cleavage rather than expression.

### 3.3. INF4E Pretreatment Enhances RISK Pathway Protective Activity

**Figure 4:**
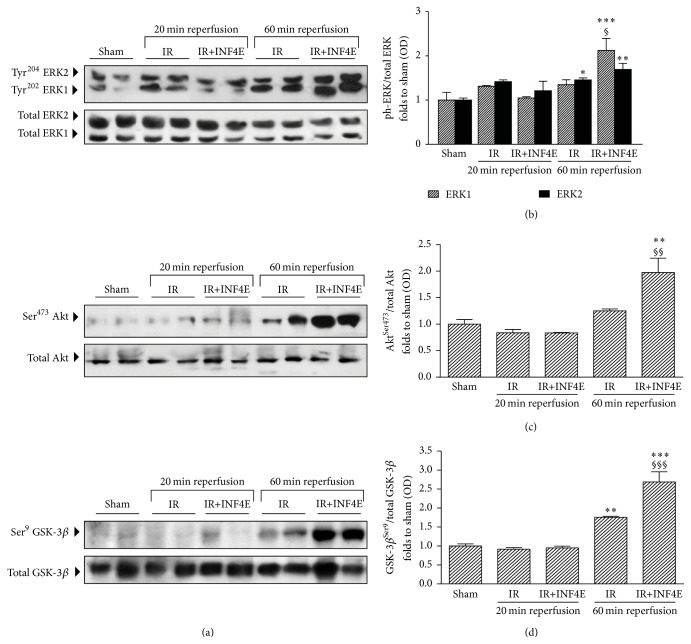
Prosurvival RISK pathway activation in the rat hearts exposed to 30 min ischemia plus 20 or 60 min reperfusion, pretreated or not with INF4E. (a) Representative Western blotting for cardiac levels of total ERK1/2 expression and Thr202/Tyr204 and Thr185/Tyr187 phosphorylation, respectively, total Akt protein expression and Ser473 phosphorylation, and total GSK-3*β* protein expression and Ser9 phosphorylation, performed on heart extracts. (b)–(d) Histograms report densitometric analysis of the phosphorylated-to-total form ratio. Data are means of 6 rats ± SEM. ^*∗*^ *P* < 0.05, ^*∗∗*^ *P* < 0.005, and ^*∗∗∗*^ *P* < 0.001 versus Sham; ^§^ *P* < 0.05, ^§§^ *P* < 0.005, and ^§§§^ *P* < 0.001 versus IR.

Very recently, a role for NLRP3 inflammasome activation in the modulation of the Reperfusion Injury Salvage Kinase (RISK) pathway has been suggested [20]. Thus, we quantified expression and activity (in terms of phosphorylation) of the key members of this pathway. After 20 min of reperfusion no modulation of the activity and/or expression level of members of the RISK pathway was recorded (Figure 4). On the contrary, the longer IR challenging induced increased phosphorylation rate of both ERK1/2 (Figures 4(a) and 4(b)) and GSK-3*β* (Figures 4(a) and 4(d)), while phosphorylation of Akt tended to increase, without reaching statistical significance, in untreated hearts exposed to either short or long IR protocol (Figures 4(a) and 4(c)). Interestingly, the INF4E pretreatment further increased the phosphorylation rate of ERK1/2, Akt, and GSK-3*β* induced by the 60 min reperfusion in untreated hearts (Figures 4(b) and 4(c)), suggesting an enhancement of the activation of this protective pathway by the pharmacological intervention.

### 3.4. INF4E Pretreatment Improves Mitochondrial Biogenesis and Energy Metabolism

**Figure 5:**
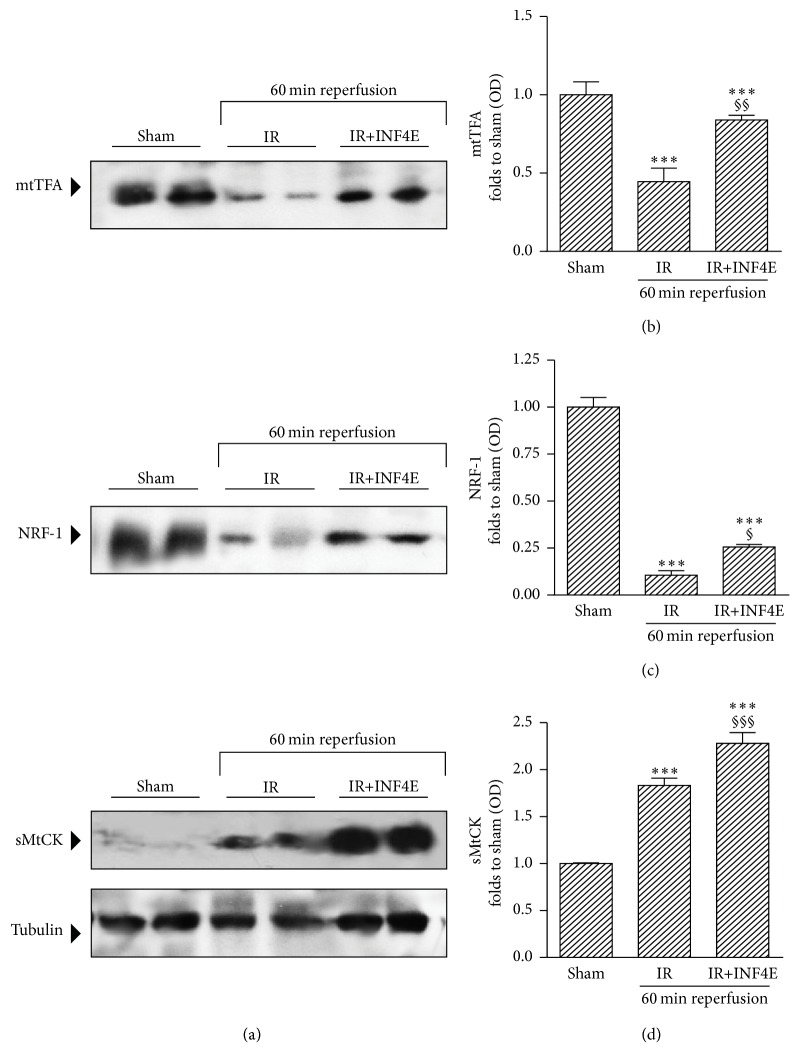
Mitochondrial biogenesis and energy metabolism evaluated in the rat hearts exposed to 30 min ischemia plus 20 or 60 min reperfusion, pretreated or not with INF4E. (a) Representative Western blotting for cardiac levels of MtTFA, NRF-1, and sMtCK performed on heart extracts. (b)–(d) Histograms report densitometric analysis normalized for the corresponding tubulin content. Data are means of 6 rats ± SEM. ^*∗∗∗*^ *P* < 0.001 versus Sham; ^§^ *P* < 0.05, ^§§^ *P* < 0.005, and ^§§§^ *P* < 0.001 versus IR.

Since mitochondrial metabolism is highly involved in the myocytes response to ischemic insult, and a cross-talk between mitochondria and NLRP3 has been described [21], we analyzed markers of mitochondrial biogenesis after 30 min ischemia and 60 min reperfusion. The MtTFA and the NRF-1 were markedly downregulated by IR. Conversely, mitogenesis was significantly preserved by INF4E pretreatment (Figures 5(a)–5(c)). A crucial physiological reaction of cardiac myocytes to oxygen deprivation is the enhancement of mitochondrial energy production, as suggested in our model by the increased expression of sMtCK after IR (Figures 5(a) and 5(d)). Intriguingly, the INF4E pretreatment further stimulated the expression of sMtCK by 25% with respect to untreated rat hearts (Figures 5(a) and 5(d)).

## 4. Discussion

The present study improves our understanding on the effects of the NLRP3 inflammasome targeting in acute myocardial infarction. Here we confirmed that myocardial IR induces transcription of all the inflammasome components in a time-dependent way, with slight effect detectable when hearts were exposed to 20 min reperfusion and robust overexpression and activation at the longest reperfusion time (60 min). These data are in agreement with previous papers showing increased expression levels of NLRP3 and procaspase-1 in the infarcted and noninfarcted areas in both cardiomyocytes and nonmyocyte cell, associated with augmentation of caspase-1 activity [13, 22]. We also previously demonstrated that an enhanced susceptibility to a myocardial ischemic insult, due to metabolic derangements, is paralleled by greater NLRP3 inflammasome activation in the heart [12]. However, the potential role of the innate immune NLRP3 protein complex as therapeutic target for cardiac infarction is ill defined. This is mainly due to the contrasting results so far obtained with targeted deletion of the inflammasome components. Few studies demonstrated that the depletion of even one of the inflammasome complex components, either the sensor (NLRP3) or the effector enzyme (caspase-1), can prevent its activation and protect the heart preventing ischemic injury and adverse cardiac remodelling [22–24]. In contrast, Sandanger et al. [20] showed that absence of NLRP3 results in increased myocardial infarct size after* in vivo* IR, whereas Jong et al. [14] concluded that NLRP3 plays no role in acute myocardial infarction due to low cardiac expression. As stated by the same authors, significant differences in the used methodologies as well as in the specific endpoint of interest may help to explain this inconsistency [25]. Besides, a discrepancy in the outcome between NLRP3^−/−^ and ASC^−/−^ mice after cardiac IR has been previously observed [13, 26], suggesting the existence of important inflammasome-independent effects related to targeted genetic deficiency. Thus, only the evaluation of small molecules able to selectively inhibit the NLRP3 inflammasome may allow ultimate elucidation of the potential of NLRP3 inflammasome as pharmacological target for therapeutic intervention in myocardial IR injury. Unfortunately, the lack of highly selective pharmacological inhibitors limits the investigation. INF4E is one of the few compounds that has been demonstrated to directly target the NLRP3 inflammasome and inhibit the ATPase activity of NLRP3 required for its activation. Although there are clear indications that this drug exerts a specific effect on NLRP3 inflammasome independently of the activating stimulus, the exact mechanism of action has still to be clarified [16]. Here we demonstrate, for the first time, that administration of the NLRP3 inflammasome inhibitor INF4E in a single dose significantly reduces infarct size, the main endpoint to target in cardioprotective studies. Moreover, the pretreatment with INF4E preserves systolic function, an index of reduced myocardial stunning. To the best of our knowledge, so far, only another small molecule acting as NLRP3 inflammasome inhibitor has been tested in models of acute myocardial injury, showing protective effects similar to those recorded in our* ex vivo* model of myocardial IR injury [27, 28]. Interestingly, this compound did not reduce infarct size at 3 h of reperfusion, while it significantly reduced infarct size at 24 h, if administered at the beginning of reperfusion, but not after 3 h reperfusion [29], thus confirming the importance of the first period of reperfusion for IR injury development and the efficacy of pharmacological strategies [5]. Here we documented significant reduction of infarct size even after 60 min of reperfusion, with INF4E administered as pretreatment. It has to be noted that our* ex vivo* model of IR injury may allow excluding the involvement of infiltrating inflammatory cells that usually may affect the reperfusion-related injury as documented in the previous studies investigating the effects of NLRP3 inhibitors when given during reperfusion [28, 29]. Thus, our data further extend the previous findings suggesting that the continuous pharmacological inhibition of NLRP3 inflammasome pathway already during the ischemic period may significantly contribute to the beneficial effects recorded at the end of reperfusion. The kinetics of the reperfusion injury may also affect the entity of the drug target expression and, thus, protection. In fact, when comparing the 20 min reperfusion and the 60 min reperfusion models we observed a progressive expression and activity of NLRP3 inflammasome complex, thus confirming previous findings on the timing of NLRP3 inflammasome formation in the heart during ischemia reperfusion [29].

Very recently, a role for NLRP3 inflammasome activation in the cardioprotective RISK pathway has been faintly suggested, but not convincingly demonstrated [20]. Here we measured the entire RISK pathway, which includes Akt and ERK1/2 activation and GSK3*β* inhibition through phosphorylation [30]. Our data demonstrate that the activation of ERK/Akt/GSK-3*β* signaling is further enhanced by pharmacological inhibition of the NLRP3 inflammasome complex and this effect may significantly contribute to its cardioprotective effects. These data confirm our previous findings, showing that pharmacological inhibition of NLRP3 inflammasome significantly potentiates the activity of the prosurvival Akt pathway [31]. Besides, the cross-talk between NLRP3 inflammasome and RISK pathways is further confirmed by the comparative analysis of the expression/activity of members of both pathways, which show robust increase only in the 60 min reperfusion model in both cases.

Although we demonstrated a direct effect of the tested compound on NLRP3 inflammasome activation, we cannot rule out the potential interactions of INF-4E with other signaling pathways, including those involved in the regulation of the expression of NLRP3 and/or activation of the RISK pathway. Thus, a further rigorous evaluation of effects of the tested compound on other signaling pathways affected by IR is needed to better elucidate its pharmacodynamics profile.

The effects of the pharmacological inhibition of NLRP3 inflammasome on the RISK pathway are closely related to those recorded on mitochondrial metabolic response. Indeed, the mitochondrial metabolic dysfunction is crucial for cardiac damage development, as both the block of mitochondrial ATP-sensitive potassium channel (mitoKATP) and the opening of the mitochondrial permeability transition pore (mPTP) are common end effectors of the IR injury. Accordingly, pharmacological strategies which aimed to open the mitoKATP and inhibit mPTP opening effectively reduce myocardial IR injury and improve cardiomyocytes energy homeostasis via modulation of the activity of members of the RISK pathway [32–35]. We and others have demonstrated that the Akt-mediated inactivation of GSK-3*β* is critical for the prevention of myocardial IR injury, being a key event in the regulation of apoptosis and the enhancement of mitochondrial biogenesis [36–38]. Besides, the use of selective GSK-3*β* inhibitors evokes protection against IR injury and promotes cell survival by limiting mPTP opening [39, 40]. In agreement with the above-mentioned observations, the INF4E-induced amplification of the RISK pathway activation here described positively impacted on mitochondrial metabolism. In fact, our data clearly demonstrate that markers of mitochondrial biogenesis, suppressed by IR, were significantly upregulated by INF4E administration. Besides, the administered compound enhanced mitochondrial energy metabolism, shown in terms of reinforced expression of the sMtCK, which is a marker of increased mitochondrial ATP production.

Unfortunately, our study does not allow identifying the specific cell types involved in NLRP3-mediated responses. Cardiomyocytes are the most prominent cell type in the heart and loss of contractile tissue is the most important consequence of a myocardial infarction. However, in cardiomyocytes, the activation on NLRP3 inflammasome evokes caspase-1 activation and pyroptosis, but not relevant release of mature IL-1*β* [22, 41]. In contrast, NLRP3 inflammasome activation in myocardial fibroblasts induces the production of large amounts of mature IL-1*β*, causing a rapid amplification of the inflammatory response [13, 23, 42]. As cardiomyocytes are crucial target for the IL-1*β* produced by resident fibroblasts, which impairs contractile function and induces apoptotic cell death, we may speculate that the beneficial effects of INF4E are due, at least in part, to an indirect improvement in cardiomyocytes functionality.

## 5. Conclusion

Taken together, our results strengthen the crucial role of NLRP3 inflammasome activation even in the early step of myocardial injury caused by IR and show, for the first time, that its pharmacological inhibition by INF4E reduced the organ injury/dysfunction. Preservation of cardiac function by INF4E is, at least in part, attributable to a cardioprotective effect mediated by the activation of the RISK survival pathways and the improvement in mitochondrial function, which, in turn, may improve cardiac function and postischemic outcome. It has to be stressed, however, that the lack of long-term evaluation as well as data on drug effects when administered during reperfusion limit the interpretation of the clinical transferability of our findings. Thus, further rigorous evaluations are needed to gain a better understanding of both efficacy and the mechanism of action of INF4E in the settings of acute myocardial infarction.

## Supplementary Material

Online Supplementary Data show a summary of the main drug data, including chemical structure, Molecular Weight, and method of synthesis. Besides, a brief report of previously published in vitro data, showing the specificity of the NLRP3 inflammasome inhibitor, are here reported. Specifically, we show results demonstrating the inhibitory effect of INF4E on pyroptotic cell death and NLRP3 ATPase and Caspase-1 activities. We also show that INF4E does not affect the activation of the Keap1-Nrf2 pathway, which is known to play a major role in cellular defense against oxidants and electrophiles.
