# Supplementary material for: Pharmacological Inhibition of NLRP3 Inflammasome Attenuates Myocardial Ischemia/Reperfusion Injury by Activation of RISK and Mitochondrial Pathways
Source: Oxid Med Cell Longev. 2016 Dec 8;2016:5271251. doi: 10.1155/2016/5271251 (PMC5178375; doi:10.1155/2016/5271251)
Supplement: Supplementary file 1 — Online Supplementary Data show a summary of the main drug data, including chemical structure, Molecular Weight, and method of synthesis. Besides, a brief report of previously published in vitro data, showing the specificity of the NLRP3 inflammasome inhibitor, are here reported. Specifically, we show results demonstrating the inhibitory effect of INF4E on pyroptotic cell death and NLRP3 ATPase and Caspase-1 activities. We also show that INF4E does not affect the activation of the Keap1-Nrf2 pathway, which is known to play a major role in cellular defense against oxidants and electrophiles. [file 5271251.f1.pdf]

Online Supplementary Data

Pharmacological inhibition of NLRP3 inflammasome attenuates myocardial ischemia/reperfusion injury by activation of RISK and mitochondrial pathways.

Raffaella Mastrocola[1], Claudia Penna[1], Francesca Tullio[1], Saveria Femminò[1], Debora Nigro[1], Fausto Chiazza[2], Loredana Serpe[2], Giuseppe Alloatti[3], Mattia Cocco[2], Massimo Bertinaria[2], Pasquale Pagliaro[1], Manuela Aragno[1], Massimo Collino[2].
[1]*Department of Clinical and Biological Sciences, University of Turin, Torino, Italy;*
[2]*Department of Drug Science and Technology, University of Turin, Torino, Italy;*
[3]*Department of Life Sciences and Systems Biology, University of Turin, Torino, Italy.*

Summary of drug data

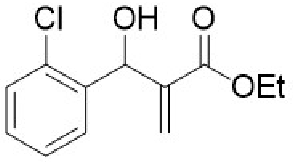

INF4E

Chemical Formula: $C_{12}H_{13}ClO_3$

Molecular Weight: 240,68 dalton

cLogP: 2.59

Methods

Syntesis: To a stirred solution of 2-chlorobenzaldheyde (4.00 g, 28.5 mmol) in a 9/1 mixture of CH_3CN / H_2O (60 mL), ethyl acrylate (9.3 mL, 85 mmol), and DABCO (3.2 g, 28.5 mmol) were added. The reaction mixture was stirred at room temperature for 7 days. The mixture was diluted with CH_2Cl_2 (30 mL), extracted with HCl 1N (3 x 30 mL), brine (30 mL), dried over anhydrous Na_2SO_4, and the solvent evaporated under reduced pressure to afford an oily residue. The crude product was purified by flash chromatography on silicagel eluting with Petroleum ether/Ethyl acetate 9:1 to afford INF4E (5.42 g, 79 %) as a colorless oil.
MS/CI (isobutano): m/z [M-H] + 241
1 H-NMR ($CDCl_3$): δ, 7.60–7.12 (m, 4H, ArH), 6.33 (s, 1H, C=CH), 5.97 (s, 1H, CHOH), 5.60 (s, 1H, C=CH), 4.20 (q, J = 7.1 Hz, 2H, CH 2 CH 3), 3.44 (s, 1H, OH), 1.25 (t, 3H, CH 2 CH 3)
13 C-NMR ($CDCl_3$): δ, 166.53, 140.89, 138.42, 132.85, 129.41, 128.98, 128.16, 127.02, 126.63, 69.23, 61.11, 14.04.
Additional details are included in a prior pubblication (Cocco M, Bertinaria M, *et al.*, Electrophilic Warhead-Based Design of Compounds Preventing NLRP3 Inflammasome-Dependent Pyroptosis. *J Med Chem.* 2014; 57(24):10366-82.

In vitro evaluation of pyroptosis: 75000 cells were plated in 48-well culture plates and treated with phorbol myristate acetate (PMA, 50 nM, 24 h; Sigma-Aldrich) to differentiate them into THP-1 cells. After 2 washing with PBS, PMA-differentiated THP-1 cells were treated with LPS (5 µg/mL, 4 h; Sigma-Aldrich) in serum-free medium. To induce cell death, ATP (5 mM, 1 h; Sigma-Aldrich) or Nigericin (10 µM, 1.5 h; Adipogen International) were used. Cell death was measured using the CytoTox 96 nonradioactive cytotoxicity assay (Promega Corporation, Madison, MI, USA), based on a colorimetric evaluation of LDH activity in supernatants. To evaluate compound's anti-pyroptotic power, cells were treated with INF4E 1h before ATP treatment at the dose range of 0.001 µM - 10 µM.

NLRP3-ATPase Activity: Human recombinant NLRP3 (0.105 µg; BPS Bioscience, San Diego, CA, USA) was incubated with INF4E (50 µM) in a buffer containing 20 mM Tris-HCl, pH 7.8, 133 mM NaCl, 20 mM MgCl2, 3 mM KCl, 0.56 mM EDTA, DMSO 0.5 % for 15 min at 37 °C. At this time point ATP (250 µm, Ultra Pure ATP) was added to the mixture, that was further incubated for 40 min at 37 °C. The NLRP3-dependent hydrolysis of ATP was determined with ADP-Glo Kinase Assay (Promega, Madison, MI, USA), a luminescent ADP detection kit.

Caspase-1 Activity: 50 U of THP-1 derived caspase-1 enzyme were incubated with INF4E (10 µM) in the reaction buffer (100 mM HEPES, 10% sucrose, 0.1% CHAPS, pH 7.5, 10 mM DTT). Caspase-1 activity was evaluated monitoring the release of pNA, a chromophore molecule derived from the caspase-1-specific substrate Ac-YVAD-pNA (N-acetyl-Tyr-Val-Ala-Asp-para-nitroanilide, 200 µM, 1 h; Enzo Biochem).

Reverse transcriptase-polymerase chain reaction (RT-PCR) analysis: Total RNA was extracted from THP-1 cells using EuroGold Trifast reagent (Euroclone, Milan Italy). Obtained RNA was reverse-transcribed with oligo(dT) primers using 8 µg of total RNA and the RevertAid First Strand cDNA Synthesis Kit (Thermo Fisher Scientific Waltham, MA, USA). PCR was of cDNA was then performed. PCR amplicons were resolved in a 5%-agarose gel by electrophoresis, and signals were quantified with densitometric analysis software (NIH Image J 1.48; National Institutes of Health, Bethesda, MD, USA).

Results

Inhibitory effect of INF4E on Pyroptotic Cell Death of THP-1 Cells

Pyroptosis is a programmed cell death implicated in the pathogenesis of auto-inflammatory diseases and in disorders characterized by inflammation. NLRP3 inflammasome dependent caspase-1 activation is an indispensable event in the pyroptotic cascade (He Y, Hara H, Núñez G, Mechanism and Regulation of NLRP3 Inflammasome Activation. *Trends Biochem Sci*. 2016. pii: S0968-0004(16)30148-7). Our results demonstrate that treatment of PMA-differentiated THP-1 cells with INF4E (10 µM; 1 h) evoked a robust decrease (80.9% ± 5.2%) in cellular pyroptosis induced by LPS priming and ATP treatment when compared to vehicle alone. The pEC50 of INF4E effects on THP1 cell pyroptosis were 6.9 ± 0.1 and 6.8 ± 0.1, when cells were exposed to ATP or Nigerin triggering, respectively

Table 2S: Effects of INF-4E on pyroptosis in PMA-differentiated and LPS-primed THP-1 cells

Pyroptosis decrease (%) 10 µM	80,9% ± 5,2%
pEC_{50} (ATP)	6.9 ± 0.1
pEC_{50} (Nigericin)	6.8 ± 0.1

Data are the mean ± SEM of at least four experiments run in triplicate.

Inhibitory effects of INF4E on NLRP3 ATPase and Caspase-1 activities

On the basis of its chemical structure and antipyroptotic efficacy, INF4E was tested on caspase-1 and NLRP3 ATPase activity to prove its inhibitory activity on the proposed drug target. Our results demonstrate that INF4E dependent inhibition of pyroptosis was accompanied by a strong reduction of both NLRP3 ATPase (35 ± 14%) and Caspase-1 (52 ± 11%) activities.

Table 2S: Effects of INF-4E on NLRP3 ATPase activity and on Caspase-1 activity.

	Inhibition of enzymatic activity
NLRP3 ATPase activity	35% ± 14%
Caspase-1 Activity	52% ± 11%

Data are expressed as percentage on inhibition of enzymatic activity vs vehicle alone and are the mean ± SEM of at least three experiments run in duplicate.

Effects of INF4E on Keap1-Nrf2 pathway activation.

To better elucidate the specificity of the NLRP3 inflammasome inhibitor, INF4E, we evaluated its effects on the activation of the Keap1-Nrf2 pathway, which is known to play a major role in cellular defense against oxidants and electrophiles. To evaluate the activation of this pathway, the levels of NFE2L2 (gene encoding for Nrf2) and HMOX1 (gene encoding for heme oxygenase-1, the main enzyme upregulated by Nrf2) were measured.

Our data show a time-dependent effect of INF4E on NFE2L2 and HMOX1 upregulation in THP-1 cells. Specifically, we demonstrated that INF4E does not affect NFE2L2 and HMOX1 expression up to 3 h incubation. The INF4E-induced upregulation reached statistically significant effect ($p<0.01$) at 4 h. As we reported that INF4E protective effects against pyroptosis can be detectable already at 1 h incubation (see Table 1S), we may claim that pyroptosis inhibition due to INF4E is not affected by INF4E-induced Keap1-Nrf2 pathway modulation. These data support our hypothesis that INF4E beneficial effects against myocardial IR injury are due to selective inhibition of NLRP3 inflammasome cascade, since INF4E exposure lasted less than 2 h in our study.

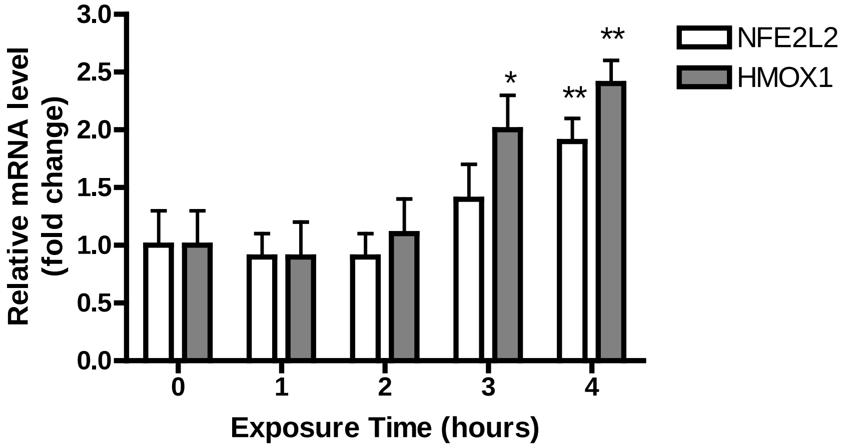

*p< 0.05; **p< 0.01vs. vehicle alone

Figure 1S: Effects of INF-4E on NFE2L2 and HMOX1 expression. THP-1 cells were exposed to INF4E (10 µM) for 1h 4h. The expression levels of marker genes encoding for Nrf2 (NFE2L2) and heme oxygenase-1 (HMOX1) were evaluated by RT-PCR analysis. Signals were densitometrically analyzed and data are expressed as mean±SEM of three determinations. The value indicated represent the ratio of the signal obtained for each sample divided by that obtained for GAPDH in the same sample.
